# Optimized deep CNN for detection and classification of diabetic retinopathy and diabetic macular edema

**DOI:** 10.1186/s12880-024-01406-1

**Published:** 2024-08-28

**Authors:** V Thanikachalam, K Kabilan, Sudheer Kumar Erramchetty

**Affiliations:** School of Computer Science & Engineering, Vellore Institute of Technology, Chennai, India

**Keywords:** Retinal Fundus Image, Diabetic Retinopathy, Diabetic Macular Edema, Discreate Wavelet transform, Artificial neural network, Adaptive Gabor Filter, Random Forest, Chicken Swarm Algorithm, Deep convolutional neural network

## Abstract

Diabetic Retinopathy (DR) and Diabetic Macular Edema (DME) are vision related complications prominently found in diabetic patients. The early identification of DR/DME grades facilitates the devising of an appropriate treatment plan, which ultimately prevents the probability of visual impairment in more than 90% of diabetic patients. Thereby, an automatic DR/DME grade detection approach is proposed in this work by utilizing image processing. In this work, the retinal fundus image provided as input is pre-processed using Discrete Wavelet Transform (DWT) with the aim of enhancing its visual quality. The precise detection of DR/DME is supported further with the application of suitable Artificial Neural Network (ANN) based segmentation technique. The segmented images are subsequently subjected to feature extraction using Adaptive Gabor Filter (AGF) and the feature selection using Random Forest (RF) technique. The former has excellent retinal vein recognition capability, while the latter has exceptional generalization capability. The RF approach also assists with the improvement of classification accuracy of Deep Convolutional Neural Network (CNN) classifier. Moreover, Chicken Swarm Algorithm (CSA) is used for further enhancing the classifier performance by optimizing the weights of both convolution and fully connected layer. The entire approach is validated for its accuracy in determination of grades of DR/DME using MATLAB software. The proposed DR/DME grade detection approach displays an excellent accuracy of 97.91%.

## Introduction

Diabetes Mellitus (DM) has reached epidemic proportions in terms of global incidence and predominance in recent years, and the study show the expected range will be in 2030 more than 360 million people who are expected to be affected by DM around the world [1]. DM is a condition in which the blood glucose level increases excessively in response to insulin insufficiency, leading to impairment of the functioning of the retina, nerves, heart and kidneys. With changes in lifestyle and dietary habits coupled with factors such as physical inactivity and obesity, DM has become more prevalent and has surpassed the status of being a disease just confined to the rich [2, 3]. DM patients are highly susceptible to developing DR, which results in abnormal retinal blood vessel growth and has a debilitating effect on vision. This progressive microvascular disorder leads to physical complications such as Diabetic Macular Edema (DME), retinal neovascularization, retinal permeability and retinal ischemia. In DR, abnormal blood vessel growth is caused by the need to supply oxygenated blood to the hypoxic retina. In addition, retinal thickening in the macular regions causes DME. It is an undisputable fact that medical treatments are more successful when diseases are discovered in their early stages.

Thereby, it is crucial to cure DR and DME in their earlier stages to prevent the serious consequence of vision loss in patients. Moreover, prior to complete blindness, there are rarely any visual or ophthalmic symptoms related to DR [4–6]. The high blood sugar levels seen in a DM patient, damages the retinal blood vessels, resulting in the leakage and accumulation of fluids such as soft exudates, hard exudates, haemorrhages and microaneurysms in the eye. The volume of these accumulated fluid defines the grade of DR, while the distance between macula and hard exudate defines the degree of DME [7]. Through early detection of DR, almost 90% of visual impairment cases are possible to be prevented. Additionally, through proper classification of DME/DR intensity, devising a suitable treatment for the DM patients is accomplished [8].

Consequently, patients with diabetes are recommended to undertake regular retinal fundal photography, in which retinal images are gathered and analysed by an ophthalmologist. Following the Airlie House DR classification, the Early Treatment Diabetic Retinopathy Study (ETDRS) group and the literature by Diabetic Retinopathy Study (DRS) group presents the classification of grades of DR using retinal fundus imaging. A conventional film camera was used in earlier days for capturing fundus images, which was later substituted by a digital camera. The fundus photography captured using Scanning Laser Ophthalmoscope (SLO) is popular nowadays [9, 10]. The manual analysis of fundus images by ophthalmologist are ineffectual in terms of high throughput screening, therefore several automatic machine learning and deep learning fundal photography-based DR/DME screening techniques are introduced [11–13].

The image processing approach is the most effective technique for identifying the grades of DME/DR owing to its promising attributes of excellent adaptability, quicker processing time and maximum reliability. In case of image processing approach, the input retinal fundus image undergoes five different stages namely pre-processing methods, segmentation, feature extraction techniques, feature selection process and efficient classification. The pre-processing technique is carried out with the intention of enhancing the quality of the input image by minimizing the noises. The mean filter is one of the prominently used filter for pre-processing owing to its effectiveness in lessening pixel intensity variations and removing redundant pixels.

However, its application is limited due to the drawback of initiating pseudo noise edges [14]. The linear filters are inept for pre-processing, since it blurs the edges and contrast of the image, while the non-linear filters such as median filter [15] and adaptive mean filter [16] are effective in minimizing the noises in the image, however on the downside, the blurring of vital and edge regions leads to information loss. Therefore, to overcome the drawbacks, DWT is used as the pre-processing technique. The accuracy of identification of grades of DR/DME is further improved with the aid of an appropriate segmentation technique, effective in accurate segmentation of the retinal vessels and lesions. The segmentation of the retinal fundus image is hindered by several obstacles such as non-uniform illumination, undefined artefacts, improper image acquisition, complex components and lesion shape variability [17].

The Fuzzy C-Means clustering methods presented [18] is a predominantly used segmentation technique in recent research work, which forms diverse clusters through image pixel division. The complex nature of this technique however prevents its wide scale implementation. Here, in this work, ANN is used for segmentation in response to its simple structure and high accuracy in segmentation. Some of the commonly used feature extraction techniques are sparse representation [19], global histogram normalization [20] and Fourier Transform [21]. However, these techniques are inept in terms of retinal vein recognition. Gabor filter is suitable for retinal vein extraction, but its application is hindered due to the difficulty experienced in parameter configuration. Hence, Adaptive Gabor Filter (AGF), that resolves the complications in parameter configuration of conventional Gabor filter is used in this work for feature extraction.

The choice of an appropriate feature selection technique significantly improves the classification accuracy of the classifier. The feature selection approaches like Maximize Relevancy and Minimize Redundancy (mRMR) and Relief operates with excellent computational efficiency but less accuracy in terms of feature selection. The Genetic Algorithm [22] is an also a commonly used approach for feature selection, but it is in efficient in handling huge input samples due to computational complexity. The neural network techniques like Recurrent Neural network (RNN) and Probabilistic Neural Network (PNN) require large training data sets and display weak interpretability. Thereby, in this work, RF is selected for feature selection in view of its implementational ease and robust generalization capability. After feature selection comes the process of classification. The machine learning based Logistic Regression [23] Classifier is an efficient technique with excellent discriminative potential, but it is incapable of solving linear problems. The CNN [24, 25] is a highly accurate technique, capable of quickly identifying and classifying any medical disorder. However, it requires large number of training images. Hence, a Deep CNN based classification is proposed in this work for the accurate classification of grades of DR/DME. Moreover, the working of the Deep CNN classifier is optimized using Chicken Swarm Algorithm (CSA).

A novel automatic DR/DME detection approach using optimized Deep CNN is proposed in this work. The different phases of the proposed image processing approach involve DWT for pre-processing, ANN for segmentation, AGF for feature extraction, RF for feature selection and finally CS optimized Deep CNN for classification. The retinal fundus images are provided as input for the proposed diagnosis model, and it is evaluated for its performance using MATLAB software.

As shown below, we provide numerous major breakthroughs and additions in this work that greatly improve our model’s efficacy and applicability for the identification of DME and DR:

- While some literature utilizes various optimization techniques, such as Genetic Algorithms or Harris Hawks Optimization, this paper uses the Chicken Swarm Algorithm (CSA) to optimize the deep CNN model, which is unique.
- The paper combines several techniques, including DWT for preprocessing, AGF for feature extraction, and RF for feature selection. While these methods have been individually used in other studies, the combination and the specific workflow are distinct.
- The novelty lies in the integrated approach combining DWT, ANN for segmentation, AGF, RF, and CSA-optimized Deep CNN for classifying the grades of DR/DME. This combination of methods aims to enhance the detection accuracy.
- The proposed method achieves a high accuracy rate of 97.91% in detecting and classifying DR/DME grades, which is presented as an improvement over existing methods.
- The paper highlights the effectiveness of using CSA to optimize the Deep CNN classifier, which is a novel application of this algorithm in this context.

## Literature study

DR and DME are two common complications of diabetes that can lead to vision loss and blindness if not detected and treated early. In recent research studies, the application of CNNs has shown promising results in the early detection and classification of DR and DME, ultimately contributing to the development of more effective and automated screening processes in diabetic eye care. Sundaram et al., [26] discusses an artificial intelligence-based approach for the detection of DR and DME. This model utilizes preprocessing, blood vessel segmentation, feature extraction, and classification techniques. It also introduces a contrast enhancement methodology using the Harris hawks optimization technique. The model was tested on two datasets, IDRiR and Messidor, and evaluated based on its accuracy, precision, recall, F-score, computational time, and error rate. This technology aims to assist in the early detection of these severe eye conditions, which are common causes of vision impairment in the working population, and it suggests a significant positive impact on the healthcare sector by enabling timely and cost-effective diagnosis.

He et al., [27] discusses a deep learning approach to classify DR severity and DME risk from fundus images. Three independent CNN’s were developed for classifying DR grade, DME risk, and a combination of both. They introduced a fusion method to combine features extracted by the CNNs, aiming to assist clinicians with real-time, accurate assessments of DR. The paper highlights the potential for automated systems to enhance early detection and treatment, and reports classification accuracy rates of 0.65 for DR grade and 0.72 for DME risk. Reyes et al., [28] discusses a system designed to classify DR and DME, which are common causes of blindness in diabetic patients. The system employs the Inception v3 transfer learning model and MATLAB digital image processing to analyze retinal images without the need for dilating drops, which can have side effects. Tested by medical professionals in the Philippines, the system showed reliable and accurate results, indicating its potential as an assistive diagnostic device for endocrinologists and ophthalmologists.

Kiruthikadevi et., [29] discusses the development and implementation of a system designed to detect and assess DR and DME from color fundus images using CNN’s. The system aims to automate the detection process to support early diagnosis and effective treatment, as substantially manual diagnosis by clinicians is not feasible at scale, particularly in resource-limited settings. The proposed two-stage approach first verifies the presence of Hemorrhages and Exudates in fundus images, and then evaluates the macular region to determine the risk of DME. The methodology includes image preprocessing to reduce noise, extraction of regions of interest focusing on the macular area, and generation of motion patterns to imitate the human visual system, all with the broader goal of contributing to the prevention of vision loss due to diabetes-related complications.

Sudha Abirami R and Suresh Kumar G [30] provides a comprehensive overview of the application of deep learning and machine learning models for the detection and classification of diabetic eye diseases, with a primary focus on DR. Various public datasets, like EyePACS and Messidor, and image preprocessing techniques are used to enhance the images before they are input into machine learning models like CNN’s. Transfer learning is emphasized as a critical technique to improve model performance, with most of the past work highlighting the need for classification of all types of diabetic eye diseases, not just DR. Despite powerful commercial AI solutions available, the review identifies a gap in affordable methods and suggests further development of computer-aided diagnostic models that are efficient and reliable for categorizing various diabetic eye conditions.

Lihteh Wu et al., [31] discusses the importance of categorizing and staging the severity of DR to provide adequate treatment and prevent visual loss. The paper emphasizes the global epidemic of diabetes mellitus and the associated risk of DR, a leading cause of blindness in the working-age population. DR is characterized by progressive microvascular changes leading to retinal ischemia, neovascularization, and macular edema. The International Clinical Disease Severity Scale for DR is highlighted as a simple and evidence-based classification system that facilitates communication among various healthcare providers involved in diabetes care without the need for specialized examinations. The scale is based on the Early Treatment of DR Study’s 4:2:1 rule relying on clinical examination.

This work [32] introduces a new framework for classifying DR and DME from retinal images. Using deep learning methods, particularly CNN’s, coupled with a modified Grey Wolf Optimizer (GWO) algorithm with variable weights, the research seeks to improve the precision and performance of the classification. This approach addresses the urgent problem of early detection and treatment of diabetic eye diseases, which are the major causes of blindness worldwide. The experimental results show that the suggested approach is an effective method for the accurate diagnosis of DR and DME, highlighting its potential in improving the diagnostic capabilities and care of patients in ophthalmology.

The paper [33] proposes a robust framework for classifying retinopathy grade and assessing the risk of macular edema in DR images. The study introduces a comprehensive approach that integrates image preprocessing, feature extraction, and machine learning algorithms to accurately classify retinal images and predict the likelihood of macular edema. By leveraging a combination of handcrafted features and deep learning techniques, such as CNN’s, the framework achieves high classification accuracy and robustness. The proposed methodology addresses the urgent need for automated and accurate diagnosis of DR, providing a valuable tool for clinicians in assessing disease severity and guiding treatment decisions. Experimental results demonstrate the effectiveness of the proposed framework in accurately classifying retinopathy grade and predicting macular edema risk, highlighting its potential for enhancing clinical workflows and improving patient outcomes in diabetic eye care.

In summary, CNN’s are a highly effective method for the classification and grading of DR and DME, with various approaches including feature reduction, attention mechanisms, and network fusion methods contributing to their success. The integration of deep learning techniques with traditional image processing methods and novel architectures has led to significant improvements in the accuracy and efficiency of diagnosing these conditions.

### Proposed system framework

**Fig. 1:**
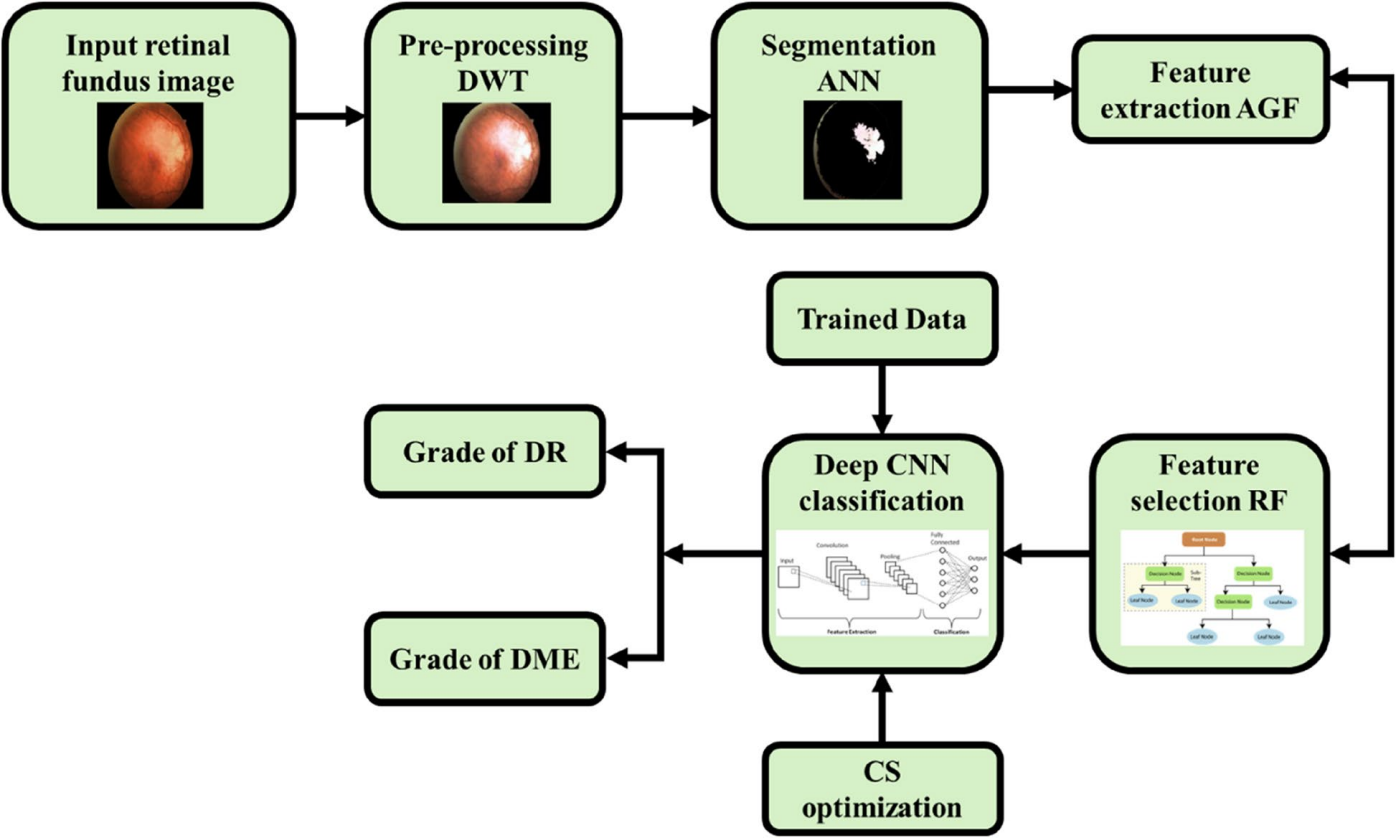
Automatic DR/DME grade detection using optimized Deep CNN architecture

The disease of DM has become a prominent disorder found in many middle aged and older generations due to the drastic unhealthy changes witnessed in food habits and lifestyle of humans. Thus, the DM is no longer considered to be the disease only confined to the rich. The person who develops DM are affected many complications among which DR and DME are the one that has direct impact over the vision. The effects of DR and DME are highly critical, since it eventually leads to a complete blindness. Through a timely accurate identification of degree of DR/DME in a diabetic patient, the condition of blindness is greatly prevented [34]. Thereby, an accurate DR/DME grade detection approach as illustrated in Fig. 1 is proposed in this work.

The proposed approach using DWT for pre-processing of the retinal fundus image. Through pre-processing, the unwanted noises that affects the retinal photography is removed and an enhanced image with uniform resolution is obtained as output. Next the pre-processed image is subjected to ANN segmentation, which is highly effective in isolation of the required region of interest. Subsequently, AGF with high reginal vein recognition capability is used for feature extraction. Moreover, the vital features that assists classification are selected among all the extracted features using the approach of RF. Finally, the degree of DR/DME is accurately detected using CS optimized Deep CNN classifier. The CSA is used for optimizing the weights of both convolution and fully connected layer, resulting in the improvement of the classification performance of Deep CNN. Moreover, the entire technique is validated in MATLAB software for ascertaining its significance in identification of DR/DME grades.

A) Preprocessing using DWT

Pre-processing is one of the crucial steps undertaken in image processing to improve the image quality and thereby enhance the accuracy of DR and DME identification. Here, the pre-processing of fundus images is done using DWT [35], which is characterized with an excellent image decomposition property. Initially the images are resized to obtain uniform resolution and increased processing speed. Then the green channel image that has vital information are extracted before undergoing histogram equalization. The resultant image with improved dynamic range and contrast are made noise free through filtering.

The fundus image is decomposed into several sub band images. At the end of every computed value in decomposition stage, the frequency resolution is twice, and the computed time resolution is halved. The products of decomposition are detail coefficients and approximation coefficients, where the latter is further decomposed into detail coefficients and values of approximation coefficients in every later level. The approximation coefficient is the first sub-band image, while the remaining coefficient are detailed coefficients, so resulting in the formation of several sub-band images. The translation parameters and discrete set of scale used in DWT are $\:\left(\tau\:=n{2}^{-m}\right)$ and $\:\left(s={2}^{-m}\right)$ respectively. The wavelet family is given as,

1 $$\:{\xi\:}_{m,n}\left(t\right)={2}^{\frac{m}{2}}\xi\:\left({2}^{m}t-n\right)$$

The $\:\:x\left[n\right]$ decomposition is given as,

2 $$\:x\left[n\right]=\sum\:_{j=1\:to\:J}\sum\:_{k\in\:U}{c}_{j,k}g\left[{n-2}^{j}k\right]+\sum\:_{k\in\:U}{d}_{J,k}{h}_{J}\left[{n-2}^{j}k\right]$$

Where the scaling and wavelet coefficients are specified as $\:{\:d}_{j,k}j=1\dots\:J$ and $\:{\:c}_{j,k}j=1\dots\:J$ respectively.

3 $$\:{\:c}_{j,k}=\sum\:_{n}x\left[n\right]{g}_{j}^{*}\left[n-|{2}^{j}k\right]$$

4 $$\:{\:d}_{j,k}=\sum\:_{n}x\left[n\right]{h}_{J}^{*}\left[n-|{2}^{J}k\right]$$

Where, the scaling sequence, wavelet and complex conjugate are expressed as $\:\:{h}_{J}\left[n-{2}^{J}k\right]$, $\:{g}_{j}\left[n-{2}^{j}k\right]$ and (*) respectively. The DWT is implemented separately for every column and row of the image. The image $\:X$ is decomposed into high frequency detail coefficients $\:\:{X}_{H}^{1},\:{X}_{V}^{1}\:and\:{X}_{D}^{1}$ and low frequency approximation coefficient $\:\:{X}_{A}^{1}$.

5 $$\:X={X}_{A}^{1}+\left({X}_{H}^{1}+{X}_{V}^{1}+{X}_{D}^{1}\right)$$

The image after $\:{N}^{th}$ level decomposition is expressed as,

6 $$\:X={X}_{A}^{N}+\sum\:_{i=1}^{N}\left({X}_{H}^{i}+{X}_{V}^{i}+{X}_{D}^{i}\right)$$

The preprocessed image is then segmented using ANN.

B) Segmentation using ANN

**Fig. 2:**
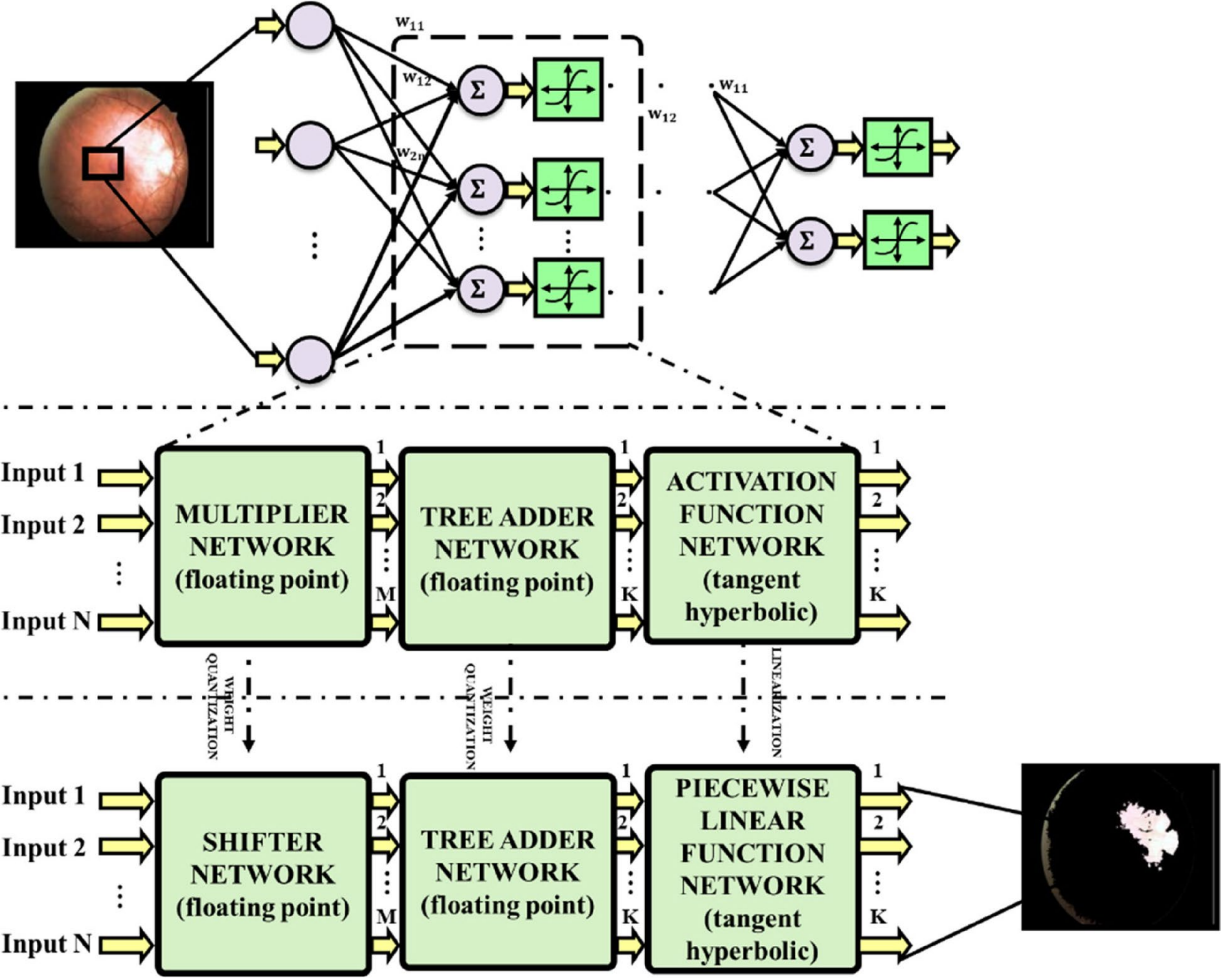
Structure of ANN

The process of segmentation is also a crucial procedure like pre-processing and is vital for the precise detection of DR and DME owing to its significant role in understanding the complex areas of interest of retinal fundus images. This image subdivision process ceases with the complete isolation of the required object of interest. In this work, ANN is used for segmentation, and it segments the pre-processed fundus images into areas and pixel groups that stands for micro aneurysms, lesions like haemorrhages, retinal blood vessels, optic disc and fovea in addition to hard and soft exudates. The ANN can impersonate the working of human brain in resolving complicated real-world problems and its structure encompasses three connected sequential layers normally called as input layer, hidden layer and output layer as presented in Fig. 2 [36].

The number of multipliers in ANN characterised with N output nodes, W hidden layer nodes and M inputs is given as,

7 $$\:EquationNumber\:of\:multiplier=M\times\:W\times\:N$$

The computational complexity of operation and calculation in each layer is reduced with the implementation of multipliers using add and shift operations rather than floating point numbers. Weights are quantized on the assumption that only a small number of shift and add operations are permitted due to the complexity of design hardware implementation. As a result, the quantization value of an original number is chosen to be the closest to it. Consider the following scenario: the maximum number of shift and add operations is 3, and the weights in the ANN are integers 0.8735 and 0.3811. The following new addition and shift operation representation may be used to represent these numbers:

8 $$\:0.8735\cong\:0.8750\:=\:{2}^{-1}+{2}^{-2}+{2}^{-3}$$

9 $$\:0.3811\cong\:0.3750\:=\:{2}^{-2}+{2}^{-3}$$

With this form, every weight is converted into a sum of power-2 integers that can be executed using shift and add operations. The ANN’s multiplier modules are therefore broken down into a few adder and shifter modules, one for each multiplier that is necessary. Even if the computational complexity is reduced by a straightforward quantization with regard to the number of power-2 operations, an error is still produced, which might be problematic in some circumstances. To solve this issue, a potential error compensation approach is shown below.

### Average quantization error reduction

Weights are quantized using only their values in the typical kind of quantization. As a result, there can be a considerable loss of accuracy due to accumulating quantization errors. Consequently, a compensating error approach is suggested [37]. There might be some accuracy decrease with each quantization. However, each image region is similar, and subsequent weight quantization can make up for the accuracy loss caused by weight quantization. By doing this, both average error and accuracy loss may be decreased. This is accomplished by distributing the generated mistake in the subsequent weight quantization, which comes after each weight has been quantized. Take the following instance into consideration. Three different weight coefficients of 0.8000, 0.4250, and 0.4050 are considered, and only three shift and add operations are permitted. It is displayed how close the closest quantized value is as shift and add number.

10 $$\:0.8000\cong\:0.7500\:=\:{2}^{-1}+{2}^{-2}\:=\:>quantization\:error\:=\:0.8000-0.7500\:=\:+0.0500$$

11 $$\:0.4250\cong\:0.3750\:=\:{2}^{-2}+{2}^{-3}=\:>quantization\:error\:=\:0.4250-0.3750\:=\:+0:0500$$

12 $$\:0.4250\cong\:0.3750\:=\:{2}^{-2}+{2}^{-3}=\:>quantization\:error\:=\:0.4250-0.3750\:=\:+0:0300$$

Consequently, the average quantization error is

13 $$\:average\:error\:=\frac{+0.0500+0.0500+0.0300}{3}=\:+0.0433$$

Diffusion of each quantization mistake during the subsequent phases of weight quantization might lower the average quantization error. In the instance of example that has.

14 $$\:0.8000\cong\:0.7500\:=\:{2}^{-1}+{2}^{-2}=\:>error\:=\:0.8000-0:0.7500\:=\:+0.0500$$

15 $$\:0.4250\underset{\Rightarrow\:}{\begin{array}{c}error\\\:\:difusion\end{array}}=0.4250+0.0500=0.4750\cong\:0.5000=\:\:{2}^{-1}=>error=0.4250-0.5000=\:-0.0750$$

16 $$\:0:4050\:\underset{\Rightarrow\:}{\begin{array}{c}error\\\:\:difusion\end{array}}\:=\:0.4050\:+\:0.0500-0.0750\:=\:0.3800\cong\:0.3750\:=\:{2}^{-2}+{2}^{-3}\:=\:>error\:=\:0.4050-0.3750\:=\:+0:0300$$

17 $$\:average\:error\:=\frac{+0.0500-0.0750+0.0300}{3}=\:+\frac{0.0050}{3}=\:+0.0016$$

The current quantization step considers all quantization faults from earlier levels. Consequently, + 0.0500 is added to current value of 0.4250. The present quantization considers the values (+ 0.0500 and 0.0750). This implies that 0.4050 is added to previous values of + 0.0500 and 0.0750. Because the prior quantization mistakes are considered in the current weight quantization in this case, the average error is lowered. The overall quantization error can be decreased using this method.

### Activation function linearization

The most popular ANN activation function is hyperbolic tangent, which has the following form.

18 $$\:\text{tanh}\left(x\right)=\:\frac{2}{1+{e}^{2x}}-1$$

Thus, a floating-point division and an exponential operation both need to be computed. It may be effective to lower the overall computation volume by linearizing and simplifying activation function. The four intervals that make up domain of tanh(x) function in this chapter are utilised to create a linear approximation function in each interval.

19 $$\:Picewise\:Linear\:Approximation\:of\:tanh\left(x\right)\:=\left\{\begin{array}{c}\begin{array}{c}\pm\:x\:\:\:\:\:\:\:\:\:\:\:\:\:\:\:\:\:\:\:\:\:\:\:\:\:\:\:\:\:\:\:\:\:\:\:\:\:\:for\:0\le\:\pm\:x<0.5\\\:\pm\:\left(0.5+\frac{(ABS\left(x\right)-0.5)}{2}\right)\:\:\:\:for\:0\le\:\pm\:x<0.5\\\:\pm\:\left(0.75+\frac{(ABS\left(x\right)-1)}{4}\right)\:\:for\:1\le\:\pm\:x<2\end{array}\\\:\pm\:\:\:\:\:\:\:\:\:\:\:\:\:\:\:\:\:\:\:\:\:\:\:\:\:\:\:\:\:\:\:\:\:\:for\:\pm\:x\ge\:2\end{array}\right.$$

With the aid of pricewise linear function, computation is accomplished leaving division and multiplication and all operations are in shift or addition form.

C) Feature extraction using adaptive gabor filter (AGF)

The AGF is used for feature extraction of the ANN segmented retinal fundus images [38]. Because it resembles the receptive field profiles in human cortical simple cells, Gabor filtering is an effective computer vision feature analysis function. Gabor filters have been effectively used by earlier academics to exploit a variety of biometric traits. A complex sinusoidal grating that is directed and modulated by a 2D Gaussian function is known as a circular AGF.

20 $$\:{G}_{\sigma\:,\mu\:,\theta\:\:}\left(x,y\right)=\:{g}_{\sigma\:}\left(x,y\right)\bullet\:\text{exp}\left\{2\pi\:j\mu\:\right(x\:cos\:\theta\:+\:y\text{sin}\theta\:\left)\right\}\:\:\:\:\:\:\:\:\:\:\:\:\:\:\:\:\:\:\:\:\:\:\:$$

Where, the term j = $\:\sqrt{-1}$ and $\:{g}_{\sigma\:}\left(x,y\right)$ refers to Gaussian envelope,

21 $$\:{g}_{\sigma\:}\left(x,y\right)=\frac{1}{2\pi\:{\sigma\:}^{2}}\:\bullet\:\text{exp}\left\{\frac{-({x}^{2}+{y}^{2}}{{2\sigma\:}^{2}}\right\}$$

The span-limited sinusoidal grating frequency $\:\:\mu\:$, the direction in the range of $\:\:{0}^{^\circ\:}-{180}^{^\circ\:}$, and the standard deviation of a Gaussian envelope which is indicated by $\:\sigma\:$. The $\:{G}_{\sigma\:,\mu\:,\theta\:}\left(x,y\right)$ term may be divided into a real part, $\:{R}_{\sigma\:,\mu\:,\theta\:}(x,y)$ and an imaginary part, $\:{I}_{\sigma\:,\mu\:,\theta\:}$ (x, y), using Euler’s formula, as illustrated in (6)–(8). In a picture, the genuine portion may be used for ridge detection while the fictitious portion is useful for edge detection.

22 $$\:{G}_{\sigma\:,\mu\:,\theta\:}\left(x,y\right)=\:{R}_{\sigma\:,\mu\:,\theta\:}\left(x,y\right)+j\bullet\:\:{I}_{\sigma\:,\mu\:,\theta\:}(\text{x},\:\text{y})$$

23 $$\:{R}_{\sigma\:,\mu\:,\theta\:}\left(x,y\right)={g}_{\sigma\:,\mu\:,\theta\:}\left(x,y\right)\bullet\:\text{cos}\left[2\pi\:\mu\:\right(x\:cos\theta\:+y\text{sin}\theta\:\left)\right]$$

24 $$\:{I}_{\sigma\:,\mu\:,\theta\:}\left(\text{x},\:\text{y}\right)={g}_{\sigma\:,\mu\:,\theta\:}\left(x,y\right)\bullet\:sin\left[2\pi\:\mu\:\right(x\:cos\theta\:+y\text{sin}\theta\:\left)\right]$$

Regions of uniform brightness, however, cause a negligible response from AGF. Direct current (DC) is what being used here. DC component is eliminated by using Eq. (9) so that Gabor filter would be insensitive to illumination:

25 $$\:{G}_{\sigma\:,\mu\:,\theta\:}\left(x,y\right)={G}_{\sigma\:,\mu\:,\theta\:}\left(x,y\right)-\frac{\sum\:_{i}^{k}=\:-k\:\sum\:_{j}^{k}\:{G}_{\sigma\:,\mu\:,\theta\:}\left(i,j\right)}{(2k+1{)}^{2}}$$

Where $\:(2k+1{)}^{2}$ is 2Dd Gabor filter size. As a result, the definition of a Gabor transform with robust illumination is given in (26), where $\:I(x,\:y)$ is an image.

26 $$\:F\left(x,y;\sigma\:,\mu\:,\theta\:\right)I\:\left(x,y\right)\bigotimes{\stackrel{\sim}{G}}_{\sigma\:,\mu\:,\theta\:}(x,y)$$

According to earlier studies, AGF-based edge identification performs best when filter parameters match the direction $\:\:\theta\:$, variance $\:\:\sigma\:$, and center frequency $\:\:\mu\:$ of input picture texture. After AGF based feature extraction, the process of feature selection RF is carried out.

D) Feature selection using random forest

The feature selection process aids in the identification of the smallest feature subset, which is pivotal to predict DR and DME with higher degree of accuracy by eliminating other irrelevant or redundant features. Thus, the choice of an effective feature selection process complements the classifier performance in identifying the DR/DME grades. The RF technique is adopted in this work for feature selection on account of its robust anti-interference and generalization capability [39]. This model aggregation-based machine learning algorithm is well suited for ill-posed and high-dimensional regression tasks. The RF when employed for feature selection, evaluates the importance score of every feature and determines their impact on the classification prediction. The RF builds decision trees using gini index and determines the final class in every tree. The impurity of node $\:\:v$ is estimated using the gini index,

27 $$\:Gini\left(v\right)=\sum\:_{i=1}^{I}{f}_{i}\left(1-{f}_{i}\right)$$

Where, the fraction of $\:class-i$ records are specified as $\:\:{f}_{i}$. For splitting the tree node $\:v$, the Gini gain information of feature $\:{\:X}_{i}$ is given as,

28 $$\:gain\left({X}_{i},v\right)=Gini\left({X}_{i},v\right)-({W}_{L}Gini\left({X}_{i},{v}^{L}\right)+{W}_{R}Gini\left({X}_{i},{v}^{R}\right)$$

Where, the right and left child node of node $\:v$ is specified as $\:{\:v}^{R}$ and $\:{\:v}^{L}$ respectively, while the node $\:v$ impurity is specified as $\:Gini\left({X}_{i},v\right)$. The child nodes are assigned with fraction of examples referred as $\:{\:W}_{R}$ and $\:{\:W}_{L}$. The splitting feature is the one that maximizes impurity reduction. The $\:gain\left({X}_{i},v\right)$ is used for calculating the importance score of $\:{\:X}_{i}$,

29 $$\:{Imp}_{i}=\frac{1}{{n}_{tree}}\sum\:_{k \epsilon S{x}_{i}}gain\left({X}_{i},v\right)$$

Where, the split nodes and ensemble size is specified as $\:k \epsilon S{x}_{i}$ and $\:{\:n}_{tree}$ respectively. The normalization of the importance score is,

30 $$\:{Imp}_{norm}=\frac{{Imp}_{i}}{{Imp}_{max}}$$

Here, the maximum importance is specified as $\:{\:Imp}_{max}$ [$\:{0\le\:Imp}_{max}\le\:1$]. The weight $\:gain\left({X}_{i},v\right)$ utilizes the importance score of preliminary RFs, thereby the penalized gini information gain is estimated as,

31 $$\:{gain}_{G}\left({X}_{i},v\right)={\lambda\:}_{i}gain\left({X}_{i},v\right)$$

The regularization level is regulated by the base coefficient of $\:{\:X}_{i}$, which is represented as $\:{\:\lambda\:}_{i} \epsilon \left[\text{0,1}\right]$.

32 $$\:{\:\lambda\:}_{i}=1-\gamma\:+\gamma\:{Imp}_{norm}$$

The weight of $\:{\:Imp}_{norm}$ is controlled by the importance coefficient represented as $\:\:\gamma\: \epsilon \left[\text{0,1}\right]$. For an $\:{\:X}_{i}$ without maximum $\:{\:Imp}_{norm}$, smaller $\:{\:\lambda\:}_{i}$ is effectuated by larger $\:\:\gamma\:$, ultimately leading to a larger penalty on $\:{\:gain}_{G}\left({X}_{i},v\right)$. In case of maximum penalty,

33 $$\:{\lambda\:}_{i}={Imp}_{norm}$$

The $$\:{\:gain}_{G}\left({X}_{i},v\right)$$ is,

34 $$\:{gain}_{G}\left({X}_{i},v\right)={Imp}_{norm}gain\left({X}_{i},v\right)$$

By injecting the normalized importance score, the Gini information gain weighting is achieved. Thus, the smallest and appropriate features are selected using RF and these features are used for enhancing the classification using CS optimized Deep CNN.

E) Classification using chicken swarm optimized deep CNN

**Fig. 3:**
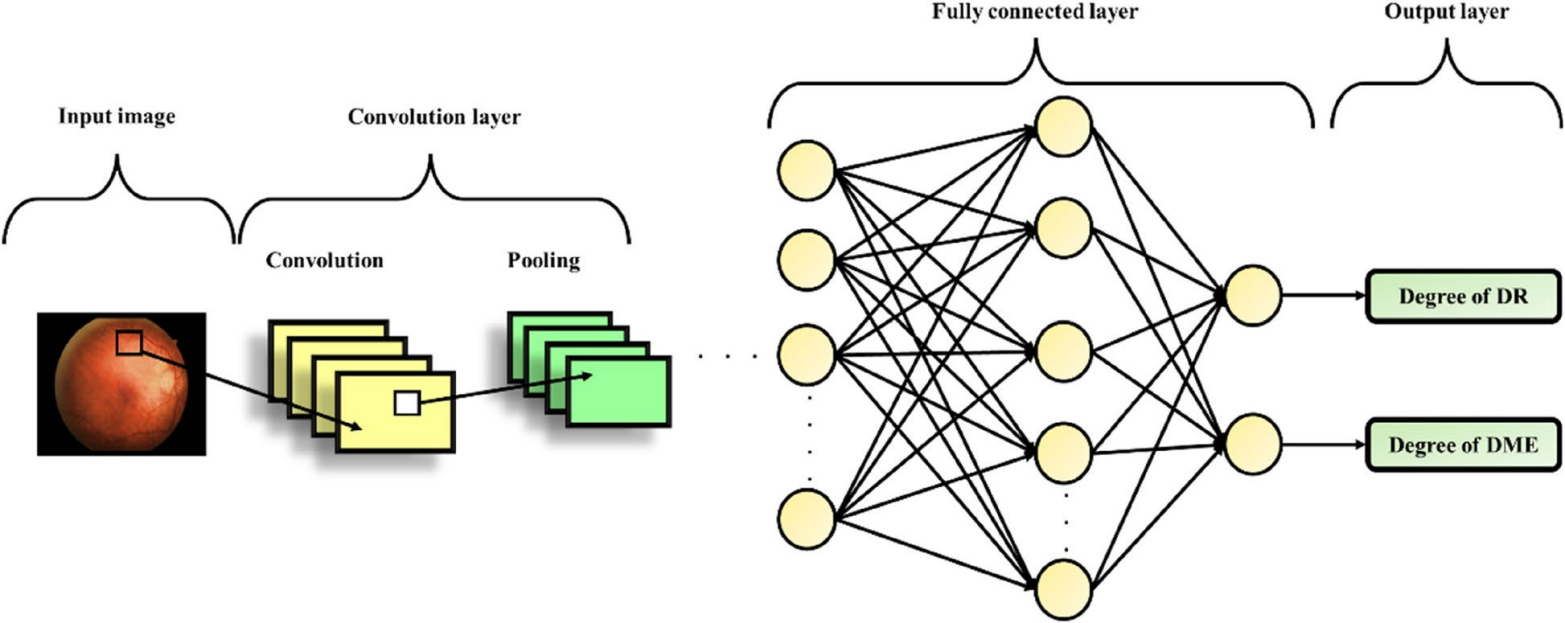
Architecture of CNN

The CS optimized Deep CNN model that are widely used for the detection are employed for classifying the grades of DME and DR. The CS algorithm is employed for optimizing the kernel values of convolution layer and optimizing the weights of the fully connected layer [40]. The features extracted using RF is provided as input to the CS optimized Deep CNN. The architecture of CNN comprises of distinct layers like convolution and pooling layers, which are grouped as modules. These modules are then subsequently followed by the fully connected layer that ultimately provides the class labels as outcomes. Modules are usually stacked on top of each other to build a deep model, which is becoming more and more popular. The structure of CS optimized Deep CNN used for the detection of DR/DME grades is given in Fig. 3.

### Convolution layers

The convolution layer observes and analyses the features of the given input and performs the operation of a feature extractor. This layer comprises of several neurons that are grouped as feature maps. Each neuron belonging to a particular feature map is connected to the other neurons in the vicinity (previous layer) using their receptive field and the filter bank, which is a trainable weight set. In this layer, the weights and inputs are combined, and the output is moved to the successive layer using a non-linear activation function. The weights of the neurons grouped in a feature map are required to be uniform, but this is not the case due to the presence of distinct feature maps with different weights, enabling the extraction of multiple features from a specific region. The$\:\:{e}^{th}$ output feature map is expressed as,

35 $$\:{x}_{e}=f\left(F{M}_{e}*{I}_{M}^{seg}\right)$$

Where, the terms $\:F{M}_{e},*and\:{I}_{M}^{seg}$ represents the $\:{\:e}^{th}$ feature map associated convolution filter, convolution operator and the input image respectively. The non-linear activation function is represented using the term $\:f(\bullet\:)$.

### Pooling layers

The pooling layers aids with attaining the spatial invariance to translation and distortion in the input. Moreover, the feature map’s spatial resolution is decreased in this layer. Initially, it is a common norm to employ average pooling layer for broadcasting the input average of small region of the image to the successive layer. The pooling layer output is given as,

36 $$\:{x}_{e}^{PL}=f\left(\sum\:_{i\in\:{M}_{j}}{x}_{e}^{PL-1}*{K}_{ij}^{PL}+{Bi}_{j}^{PL}\right)$$

Where, down sampling layer and the convolution layer are specified as $\:PL-1$ and $\:PL$ respectively. The input features of down sampling layer are represented as $\:\:{x}^{PL-1}$, while the additive bias and kernel maps of the convolution layer is specified as $\:\:{Bi}^{PL}$ and $\:{\:K}_{ij}$ respectively. The input map selection is referred as $\:{\:M}_{j}$, the output and input are indicated as $\:\:i$ and $\:\:j$ respectively. The crucial element of a field is chosen using max pooling.

### Fully connected layers

**Fig. 4:**
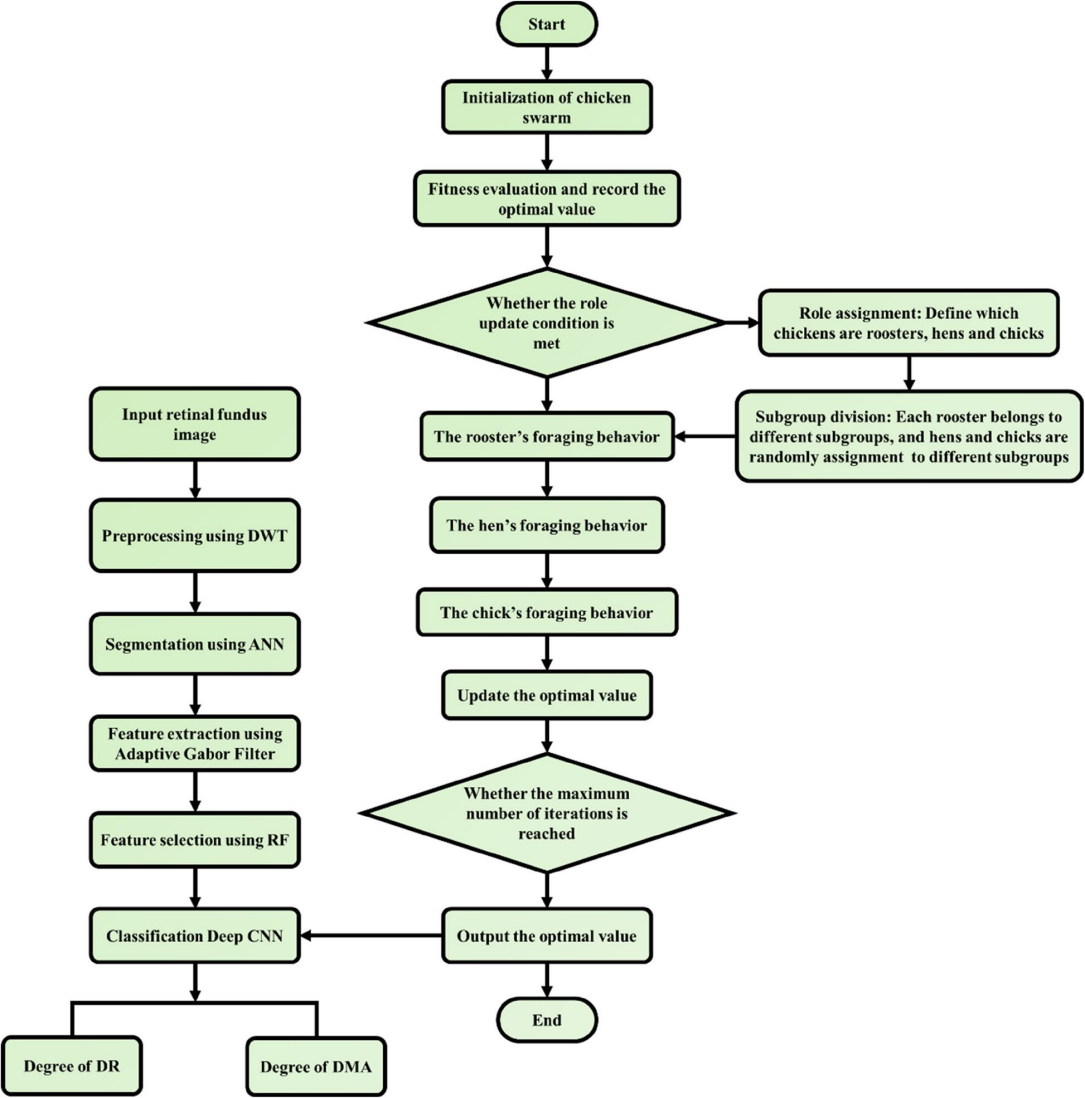
Flowchart of CS optimized Deep CNN

Several convolution and pooling layers are stacked with one another to obtain optimal feature representation. These feature representations are fully analysed by the fully connected layer to accomplish operation of high-level reasoning. The accuracy of the Deep CNN is further improved with the aid of CS optimization. The flowchart of CS optimized Deep CNN for identification DR/DME grades is shown in Fig. 4.

### Chicken swarm (CS) optimization

The CS optimization algorithm enhances the classification accuracy of the Deep CNN through optimization of the fully connected layer and convolution layer. The characteristic traits of a chicken swarm that encompasses roosters, chicks and hens forms the basis of this algorithm. The rules associated with this algorithm is given as:

- The rooster is the head of a chicken swarm, which comprises of numerous chicks and hens.
- The fitness value of the chicken determines its individuality and aids in distinguishing itself from the others. The chief rooster is the one with the best fitness value, while chicks are the ones with worst fitness value. The rest are termed as hens and a casual mother-child relationship is created between the chicks and hens.
- After several steps, each of their status gets updated.

The rooster guides the others in search of their food, while the chick forages for its food by staying in the vicinity of their mothers. In a dimensional space (DS), at a time step $\:ts$, the positions of the N virtual hens are represented as,

37 $$\:{A}_{m,n}^{ts}\left(m\in\:\left[1,\dots\:,N\right],n\in\:\left[1,\dots\:,DS\right]\right)$$

Where, the mother hens, the chicks, hens and roosters are represented using the terms $\:NM,\:NC,\:NHl$ and $\:NR$ respectively. The chance of obtaining the food is more for the rooster with best fitness value.

38 $$\:{A}_{m,n}^{ts+1}={A}_{m,n}^{ts}*(1+Randn(0,{\sigma\:}^{2}\left)\right)$$

39 $$\:{\sigma\:}^{2}=\left\{\begin{array}{c}1,\:if\:f{v}_{m}\le\:f{v}_{1}\\\:exp\left(\frac{\left(f{v}_{1}\le\:f{v}_{m}\right)}{\left|f{v}_{m}\right|+\epsilon\:}\right),\:otherwise,\:l \epsilon \left[1,N\right],l\ne\:m\end{array}\right.$$

Where, the fitness value associated with A is specified as $\:fv$, the rooster index is specified as$\:\:l$, the smallest constant used for evading the zero-division error is specified as $\:\:\epsilon\:$ and the gaussian distribution with SD $\:{\:\sigma\:}^{2}$ and mean 0 is represented as $\:Randn(0,{\sigma\:}^{2})$.

40 $$\:{A}_{m,n}^{ts+1}={A}_{m,n}^{ts}+S1*Rand*\left({A}_{ro1,n}^{ts+1}={A}_{m,n}^{ts}\right)+S2*Rand*({A}_{ro2,n}^{ts+1}={A}_{m,n}^{ts})$$

41 $$\:S1=exp\left(\left({fv}_{m}-{fv}_{ro1}\right)/\left(abs\left(f{v}_{m}\right)+\epsilon\:\right)\right)$$

42 $$\:S2=exp\left(\left({fv}_{ro2}-{fv}_{m}\right)\right)$$

43 $$\:{A}_{m,n}^{ts+1}={A}_{m,n}^{ts}+FL*({A}_{a,n}^{ts}={A}_{m,n}^{ts})$$

Where, a random number between [0,1] is specified as $\:\:Rand$. The randomly selected index from the swarm and the rooster index is represented as $\:\:ro2 \epsilon [1,\dots\:,N]$ and $\:ro1 \epsilon [1,\dots\:,N]$ respectively. Furthermore, $\:f{v}_{m}>f{v}_{ro1}$ and $\:f{v}_{m}>f{v}_{ro2}$, hence $\:\:S2<1<S1$. The probability of the chick staying nearby its mother is specified using the term FL, which lies between [0, 2].

### Results and discussion

The proposed automatic DR/DME grade detection model was confirmed for its effectiveness by executing in MATLAB. The dataset having 2072 high resolution retinal fundus images is collected from MESSIDOR [41] to assess the performance of research work proposed under CS optimized Deep CNN based diagnostic technique. Among the gathered 2072 image samples, 1402 samples belong to healthy people without diabetic condition, while 520 samples belong to diabetic patients having DR/DME. A total of 150 retinal fundus images is considered as testing data. The overall details of the selected dataset are tabulated in Table 1.

**Table 1:** Dataset details

DATA	TESTING		TRAINING	
	DR/DME INFECTED	HEALTHY	DR/DME INFECTED	HEALTHY
**SIZE**	75	75	520	1402

**Fig. 5:**
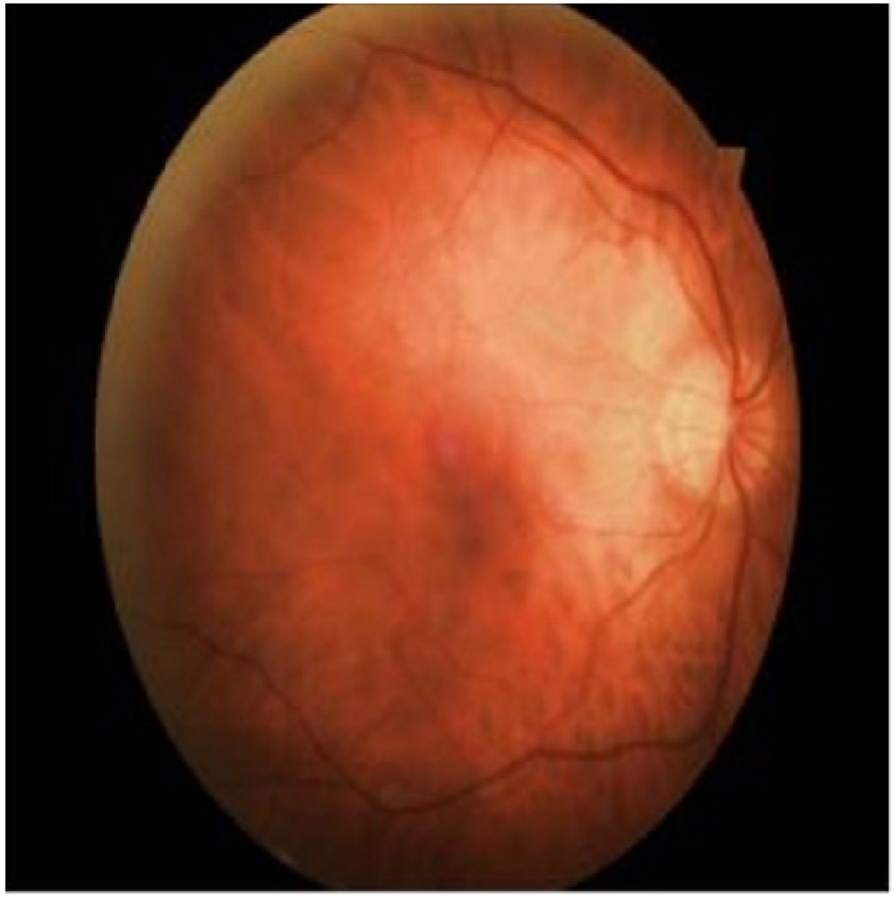
Input Image

**Fig. 6:**
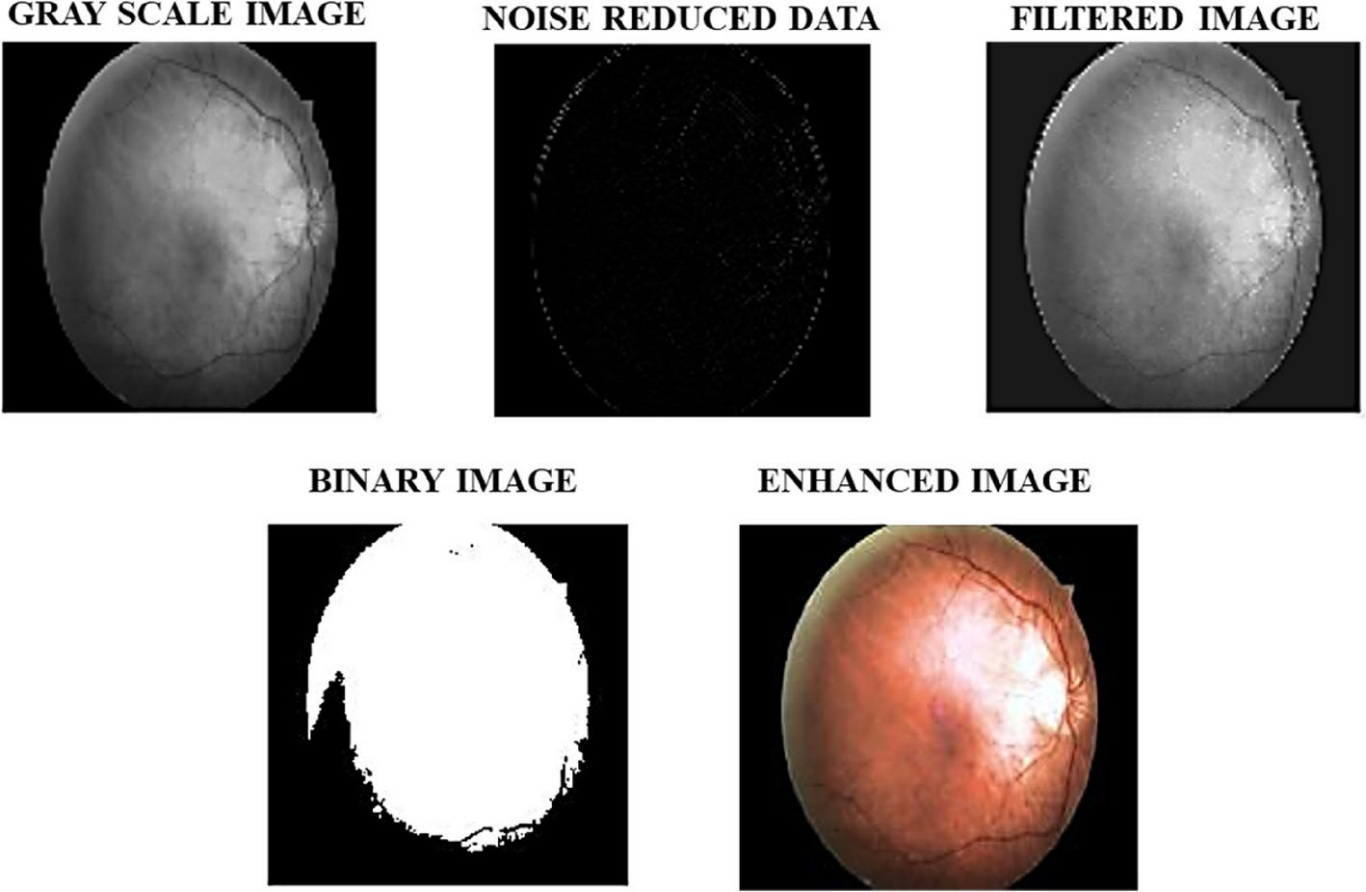
Stages of Pre-processing

The provided input retinal fundus image seen in Fig. 5, undergoes the process of pre-processing initially. The several stages involved in pre-processing is displayed in Fig. 6. The images are resized in view of supporting a uniform resolution. Then the resized input image undergoes gray scale conversion, noise reduction and filtering to obtain a pre-processed retinal fundus image of enhanced quality. In addition to obtaining a pristine noise-free image, the DWT based pre-processing also aids with reducing the processing time required for the execution of the entire technique.

The DWT pre-processing is compared against prominent techniques including the filer methods such as Mean filter, Median filter, Wiener filter and Hilbert Transform in terms of Root Mean Square Error (RMSE), Peak Signal to Noise Ratio (PSNR), Structural Similarity Index (SSIM) and Mean Square Error (MSE). The results obtained are taken for comparison in Table 2.

**Table 2:** Pre-processing techniques comparison

Filters Parameters	Mean	Median	Weiner	Hilbert Transform	DWT
**RMSE**	15.1981	13.9634	12.5763	11.2873	**7.1854**
**PSNR**	24.1954	24.9687	26.7643	27.2187	**29.6749**
**SSIM**	0.5874	0.6348	0.7734	0.8265	**0.9654**
**MSE**	119.7654	106.8434	90.2463	84.7639	**44.1327**

On analyzing the observations given in Table 2, it is concluded that the DWT performs better than all the other commonly used pre-processing techniques. Thus, the DWT technique is successful in its role of enhancing the accuracy of the proposed automatic DR/DME diagnostic system.

**Fig. 7:**
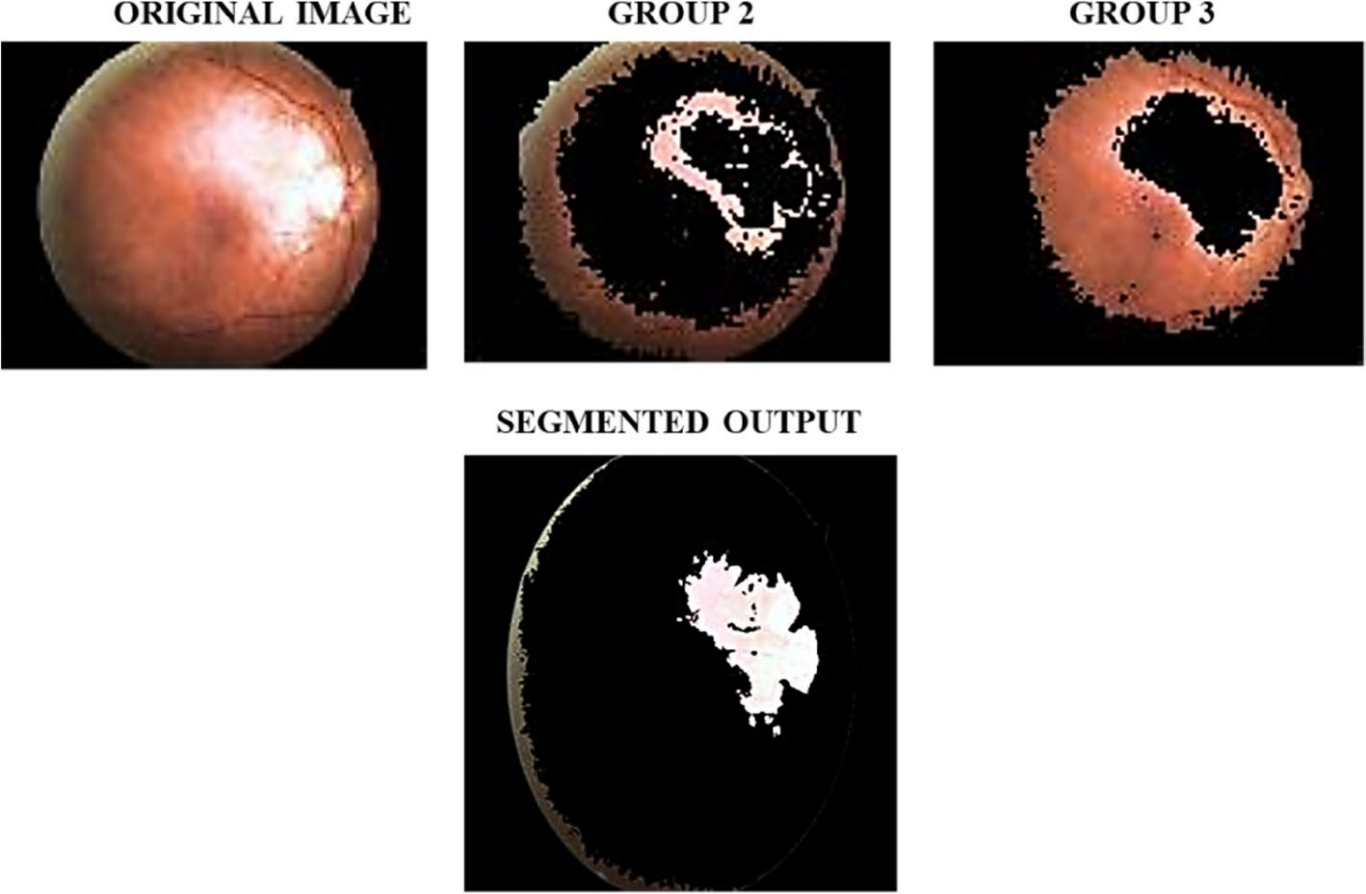
Segmentation using ANN outputs

**Fig. 8:**
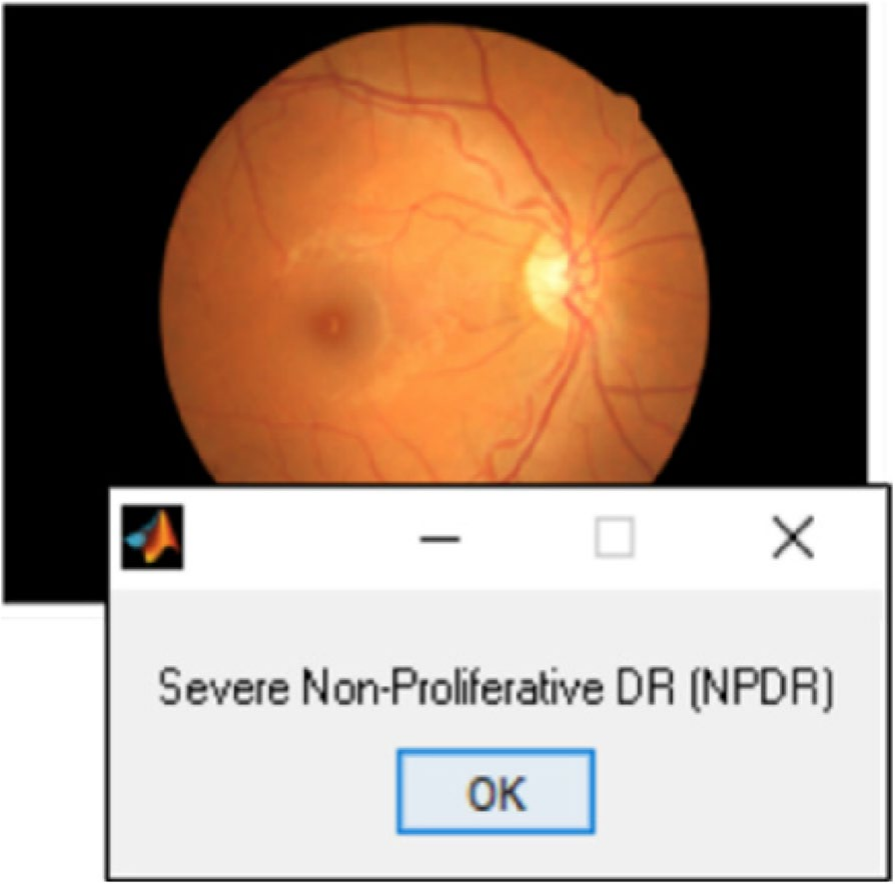
CS optimized Deep CNN classifier output

The output obtained using ANN based segmentation is provided in Fig. 7. From the obtained segmented retinal image, it is noted that the ANN is capable of accurately segmenting lesions affecting the eyes. Moreover, it is also seen that the ANN is effective in accurate segmentation of the DR/DME affected regions without compromising the image clarity. The different grades of DR are Proliferative DR, Severe Non-Proliferative DR (NPDR), Moderate NPDR and mild NPDR. Moreover, the DME is categorized in to three different grades namely mild DME, moderate DME and severe DME. So, the final classified output of the CS optimized Deep CNN classifier is shown in Fig. 8.

**Fig. 9:**
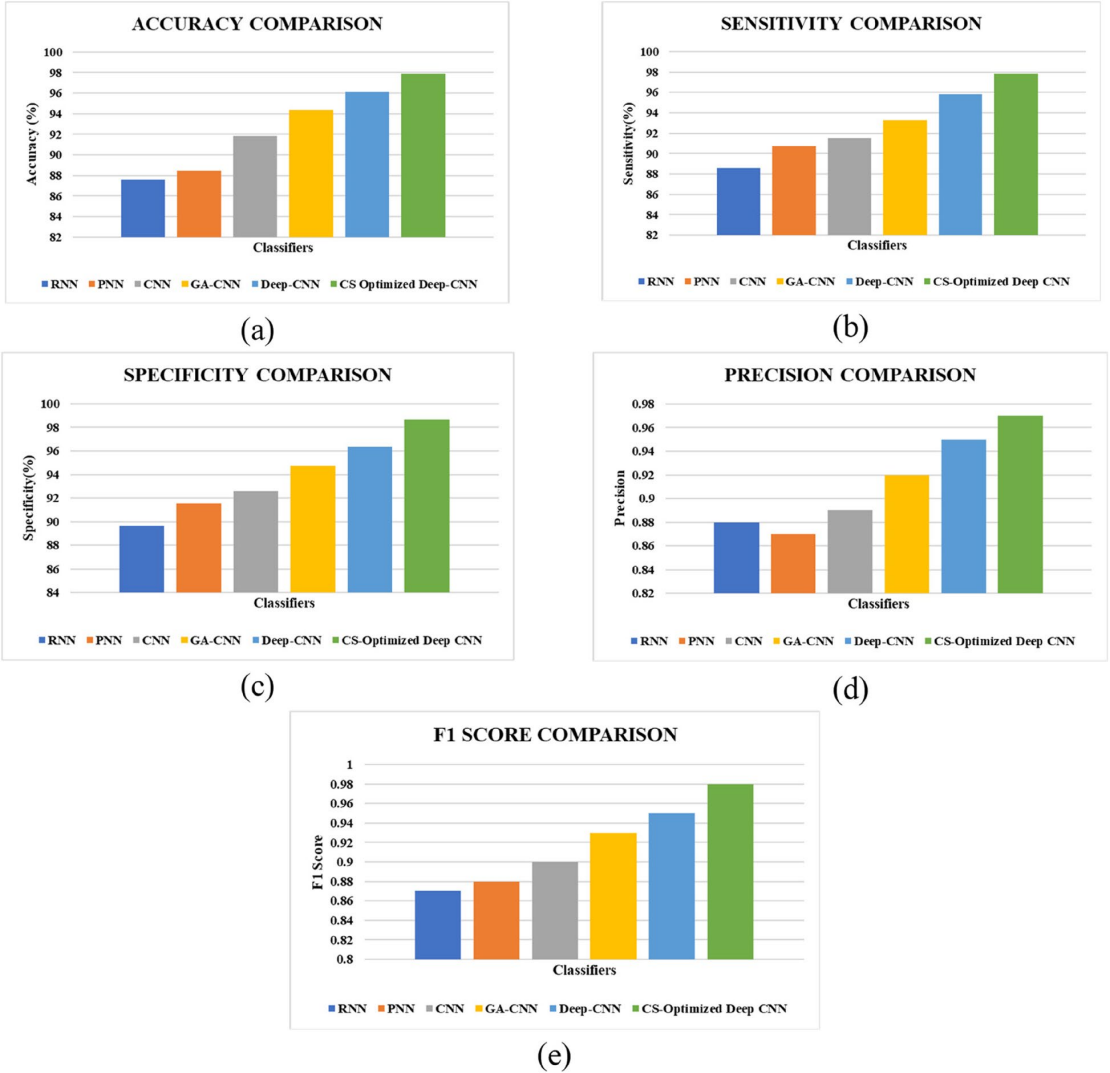
Classifier comparison in terms of (**a**) Accuracy (**b**) Sensitivity (**c**) Specificity (**d**) Precision and (**e**) F1 Score

As seen in Fig. 8, the Deep CNN accurately classifies the retinal fundus image as Severe NPDR condition. The influence of CS optimized CNN in classification is verified by comparing with the existing classifier techniques and the concerned results are tabulated in Table 3 and is also graphically represented in Fig. 9. The developed CS optimized Deep CNN has an enhanced accuracy of 97.91, sensitivity of 97.82%, specificity of 98.64%, Precision value of 0.97 and F1 score of value 0.98. Moreover, it is also noted that the CSA is effective in improving the overall performance of Deep CNN.

**Table 3:** Classifier comparative analysis

Performance Metrics Classifiers	Accuracy	Sensitivity	Specificity	Precision	F1-Score
RNN	87.62	88.57	89.65	0.88	0.87
PNN	88.43	90.76	91.54	0.87	0.88
CNN	91.85	91.49	92.56	0.89	0.90
GA-CNN	94.37	93.27	94.75	0.92	0.93
Deep CNN	96.11	95.81	96.37	0.95	0.95
CS Optimized Deep CNN	**97.91**	**97.82**	**98.64**	**0.97**	**0.98**

To assess the effect of the Random Forest feature selection procedure on the functionality of our model, we conducted an ablation study. The findings projected in Table 4 showed that adding feature selection increased the accuracy of the model from 93.85 to 97.91%, along with gains in precision, recall, and F1-score. This proves how well the feature selection process works to improve the model’s ability to correctly categorize the various grades of diabetic macular oedema (DME) and diabetic retinopathy (DR), underscoring the crucial role that feature selection plays in the overall performance of the classification process.

**Table 4:** Quantitative results from ablation study

Metric	With Feature Selection (RF)	Without Feature Selection
Accuracy	97.91%	93.85%
Precision	97.45%	92.30%
Recall	98.30%	94.15%
F1-Score	97.87%	93.21%

Recent discoveries in deep learning and medical imaging, such as Zhang et al. [42] and Zhang et al. [43], have shown the usefulness of region-based integration-and-recalibration networks for nuclear cataract categorization for AS-OCT images. These investigations emphasize the increasing significance of advanced image processing methods in raising diagnostic precision, as does the work of Xiao et al. [44], who presented a multi-style spatial attention module for cortical cataract classification.

In contrast with existing research, which mainly concentrates on AS-OCT pictures, our study improves feature extraction from retinal images by using CNNs in conjunction with Discrete Wavelet Transform (DWT). To further set our method apart, we also used the Chicken Swarm Algorithm (CSA) for model weight optimization. Our strategy provides a unique combination of DWT and CSA, exceeding the performance metrics stated in the referenced publications, which focus on attention mechanisms and recalibration.

Furthermore, our results highlight the potential of deep learning methods in real-time clinical settings, especially in automated DR and DME detection, which hasn’t been thoroughly studied with the attention mechanisms employed in existing studies, as far as we came to know. This demonstrates how innovative our methodology is in bringing these approaches to a new setting in medical imaging and advances the area of automated medical diagnosis.

## Conclusion

An automatic DR/DME grade detection approach using optimized Deep CNN is introduced in this article. The rise seen in patients affected by DM in recent times has in turn resulted in an increased risk of early age blindness because of DR and DME. Thereby, the proposed work has an impact in aiding with the earlier detection of this serious medical condition. Through prompt detection and proper treatment, a substantial number of DM patients are saved from a potential sightless dark future. In this approach, the input retinal fundus images are initially pre-processed using DWT, resulting in the deliverance of noise-free sharp contrast retinal images. Then with the application of ANN, the exact region of interest is found and segmented. The vital features that support effective classification is obtained using AGF, while RF is used as the feature selection technique in this work. Ultimately, the grades of DR/DME are identified using CS optimized Deep CNN classifier. The entire approach is evaluated for its accuracy using MATLAB software and from the derived results, it is concluded that the CSA is successful in improving the classification accuracy of the Deep-CNN classifier. The proposed automatic DR/DME grade detection technique works with an outstanding accuracy of 97.91%.

## Data Availability

IDRiR Dataset: https://ieee-dataport.org/open-access/indian-diabetic-retinopathy-image-dataset-idrid. Messidor Dataset: https://www.adcis.net/en/third-party/messidor/.

## Abbreviations

DR: Diabetic Retinopathy
DME: Diabetic Macular Edema
DWT: Discreate Wavelet Transform
ANN: Artificial Neural Network
AGF: Adaptive Gabor Filter
RF: Random Forest
CNN: Convolutional Neural Network
CSA: Chicken Swarm Algorithm
DM: Diabetes Mellitus
SLO: Scanning Laser Ophthalmoscope
mRMR: Maximize Relevancy and Minimize Redundancy
RNN: Recurrent Neural Network
PNN: Probabilistic Neural Network
GWO: Grey Wolf Optimizer
RMSE: Root Mean Square Error
PSNR: Peak Signal to Noise Ratio
MSE: Mean Square Error
SSIM: Structural Similarity Index
